# Supplementary material for: Wildfire-dependent changes in soil microbiome diversity and function
Source: Nat Microbiol. 2022 Aug 25;7(9):1419–30. doi: 10.1038/s41564-022-01203-y (PMC9418001; doi:10.1038/s41564-022-01203-y)
Supplement: Supplementary file 1 — Supplementary Figs. 1–9, Tables 1–4, Information about Supplementary Data files, and Notes 1–8. [file 41564_2022_1203_MOESM1_ESM.pdf]

nature portfolio

https://doi.org/10.1038/s41564-022-01203-y

Supplementary information

Wildfire-dependent changes in soil microbiome diversity and function

In the format provided by the authors and unedited

Supplementary information for

Wildfire-dependent changes in soil microbiome diversity and function

Amelia R. Nelson, Adrienne B. Narrowe, Charles C. Rhoades, Timothy S. Fegel, Rebecca A. Daly, Holly K. Roth, Rosalie K. Chu, Kaela A. Amundson, Robert B. Young, Andrei S. Steindorff, Stephen J. Mondo, Igor V. Grigoriev, Asaf Salamov, Thomas Borch, Michael J. Wilkins

Table of contents

Supplementary Figures 1-9	2-10
Supplementary Table 1	11
Supplementary Table 2	12
Supplementary Table 3	13
Supplementary Table 4	14
Supplementary Data Information	15
Supplementary Notes 1-8	16-22
References	23-26

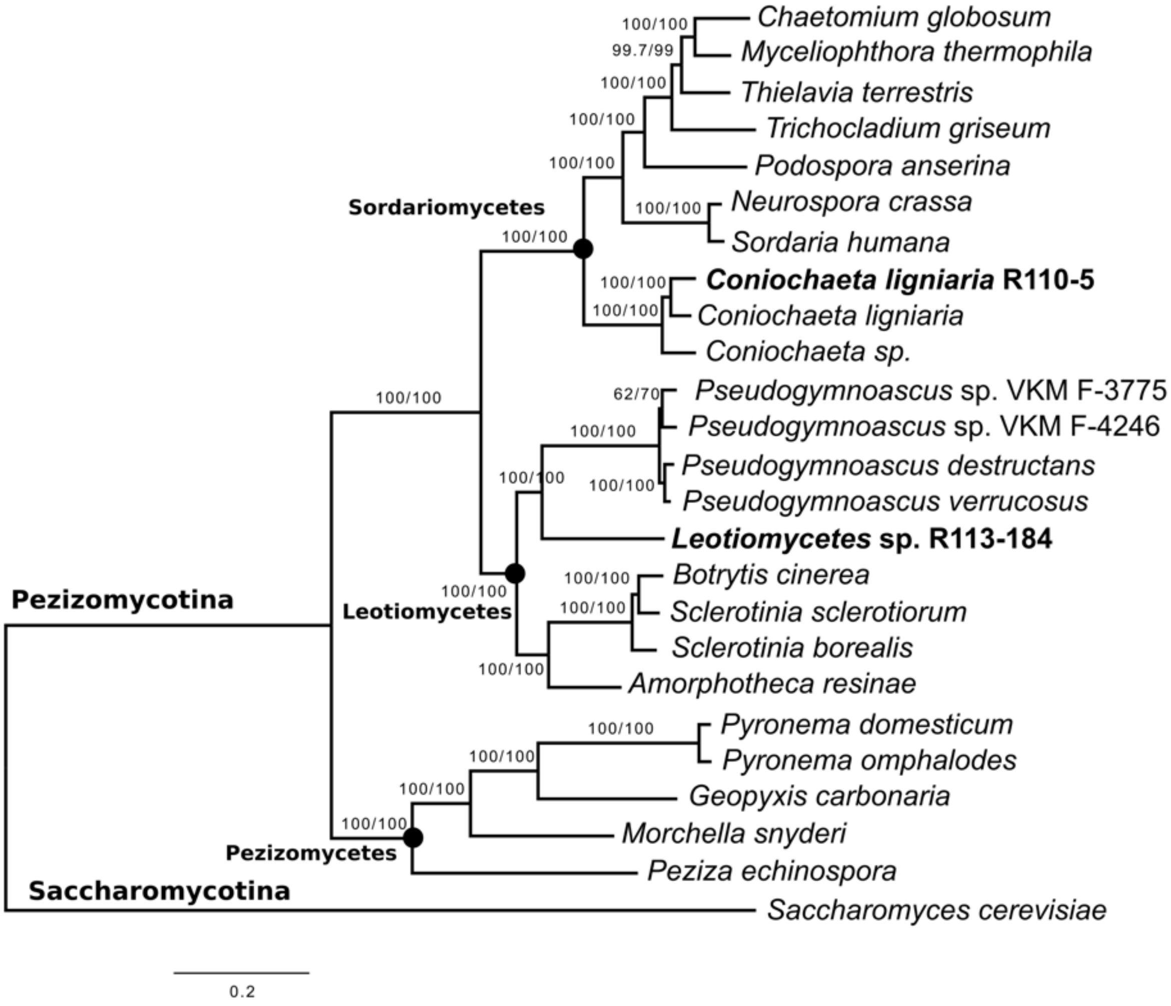

Figure S1 Fungal phylogenetic tree. Maximum likelihood tree showing the phylogenetic relationship of the 2 fungal MAGS (bold) based on 867 single-copy orthogroups.

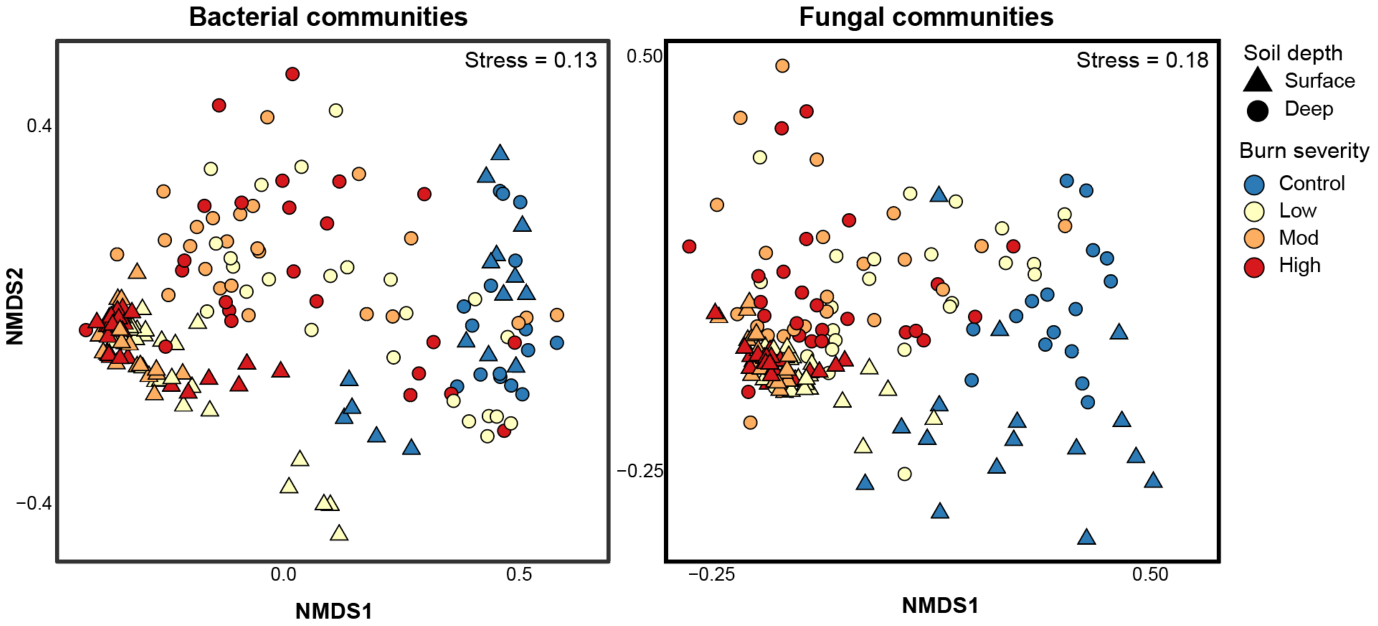

Figure S2 Broad bacterial and fungal community shifts with burn severity. Non-metric multidimensional scaling (NMDS) ordination of all bacterial (left) and fungal (right) communities shaped by soil depth and colored by burn severity.

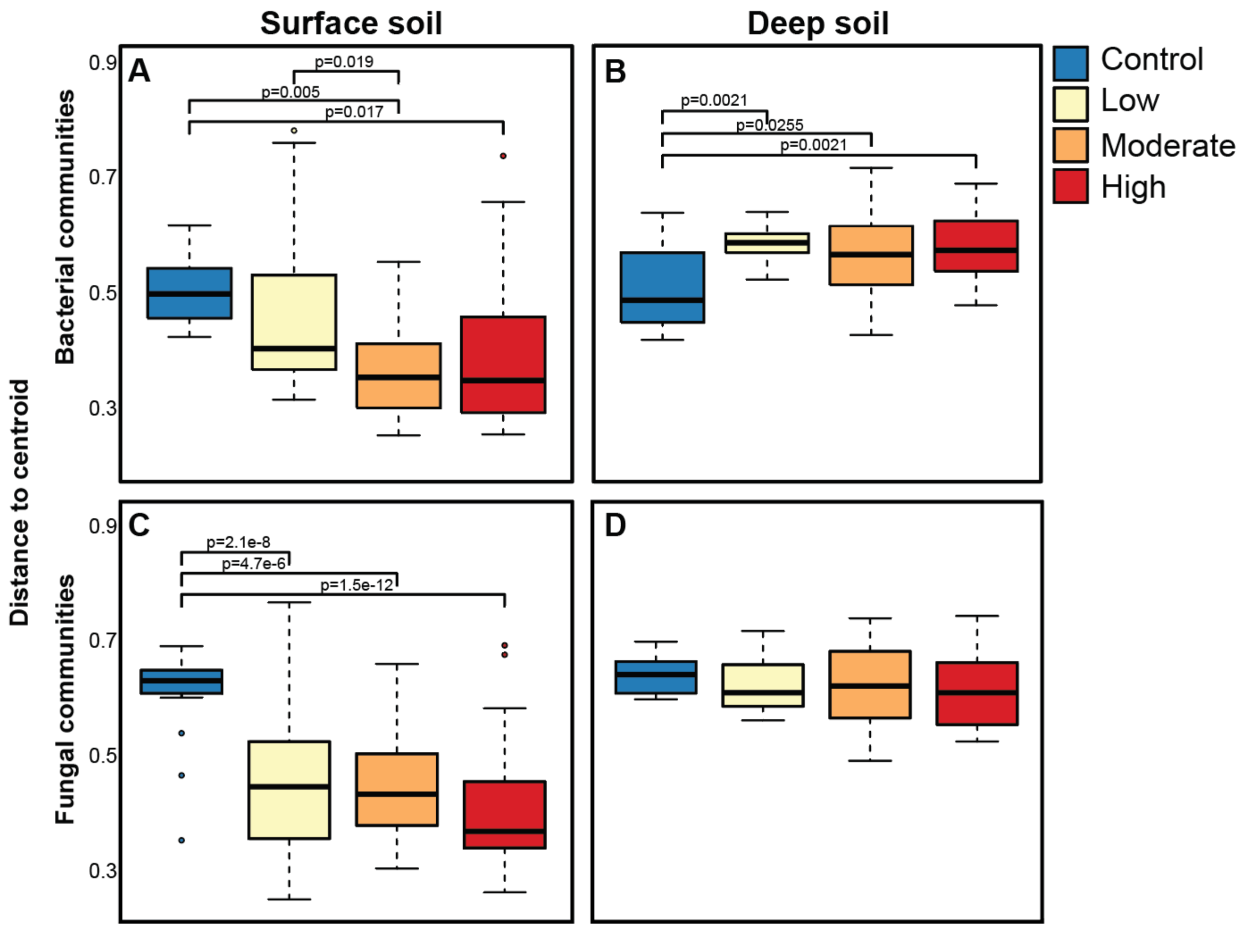

Figure S3 The impact of burn severity on surface and deep soil communities differs. Distance to centroid calculations of the NMDS ordinations of surface (A, C) and deep (B, D) soil bacterial (A, B) and fungal (C, D) communities by burn severity (n = 16 for control S and D, n = 24 for low, moderate, and high severity-impacted S and D samples). P-values are indicated if comparisons are significantly different (pairwise t-test, $p<0.05$) between conditions. The lower and upper hinges of the boxplots represent the 25th and 75th percentile and the middle line is the median. The upper whisker extends to the median plus 1.5x interquartile range and the lower whisker extends to the median minus 1.5x interquartile range.

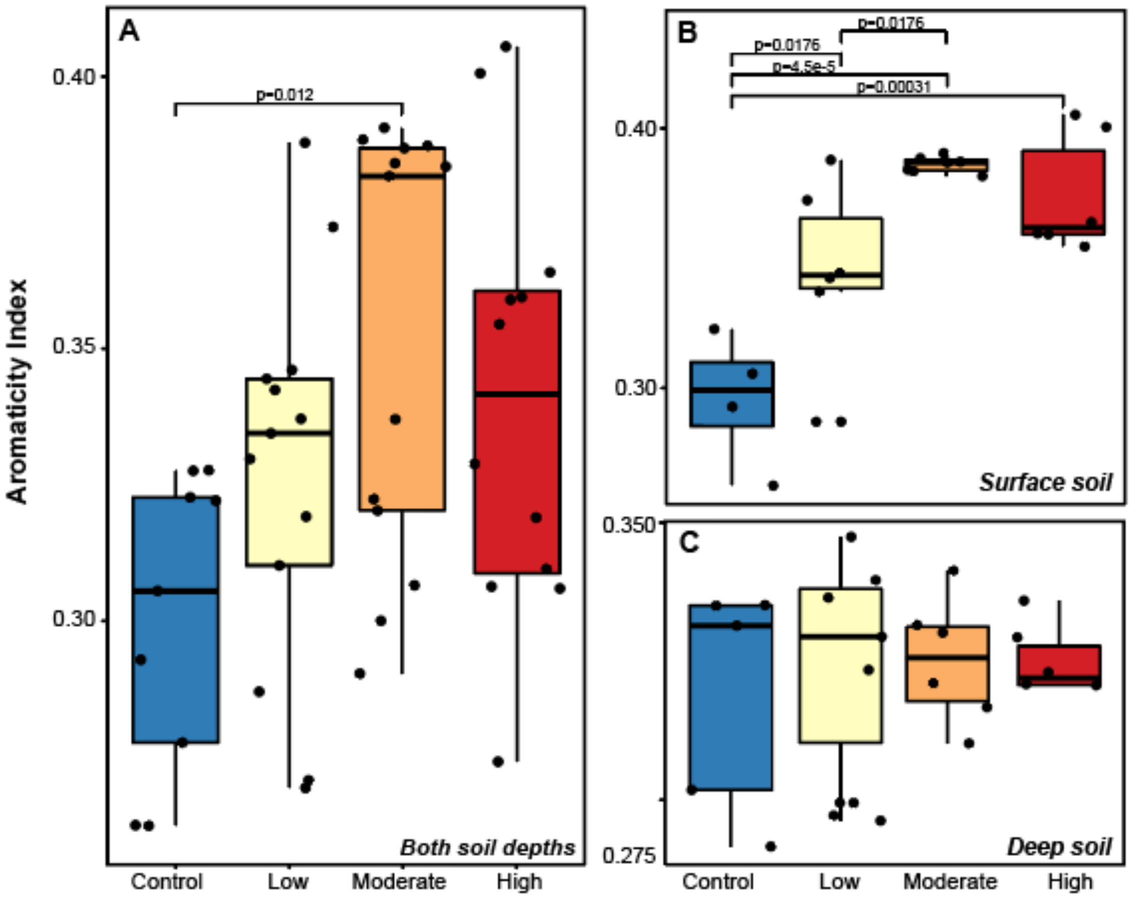

Figure S4 DOM aromaticity index trends across soil depths. Aromaticity index of DOM pools across burn severity conditions for all samples combined (A; *n* = 9 for control, 12 for low, 11 for moderate, and 12 for high severity), and shallow (B; *n* = 4 for control, 6 for low, 5 for moderate, and 6 for high severity) and deep (C; *n* = 5 for control, 6 for low, moderate, and high severity) samples. P-values are shown if there is significant differences between conditions (pairwise t-test, $p<0.05$). The lower and upper hinges of the boxplots represent the 25th and 75th percentile and the middle line is the median. The upper whisker extends to the median plus 1.5x interquartile range and the lower whisker extends to the median minus 1.5x interquartile range. Data points represent outliers.

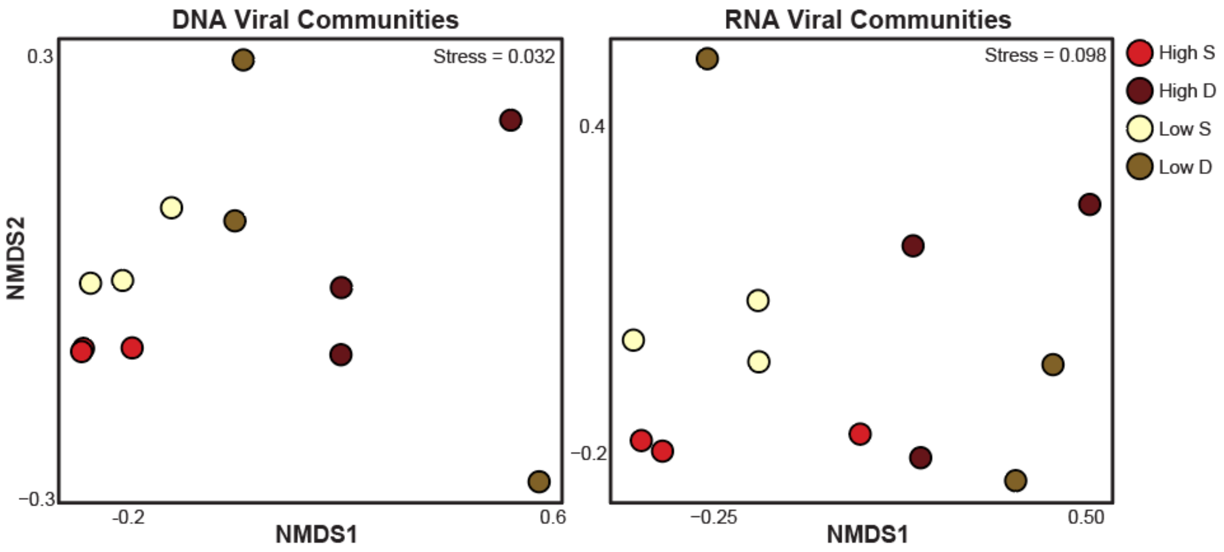

Figure S5 DNA and RNA viral community dynamics. NMDS of DNA (left) and RNA (right) vMAG abundance across all four conditions.

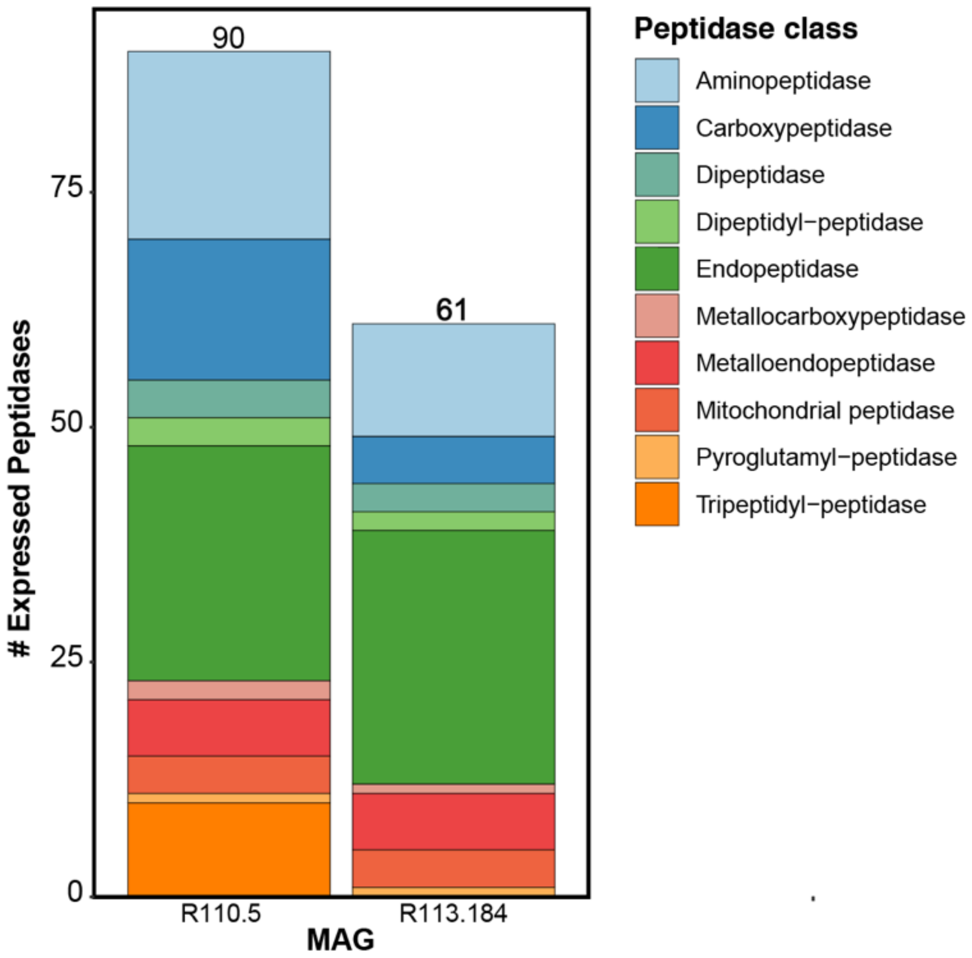

Figure S6 Peptidase expression prevalent in fungal MAGs. The diversity of all peptidases expressed by each fungal MAG across all 4 conditions.

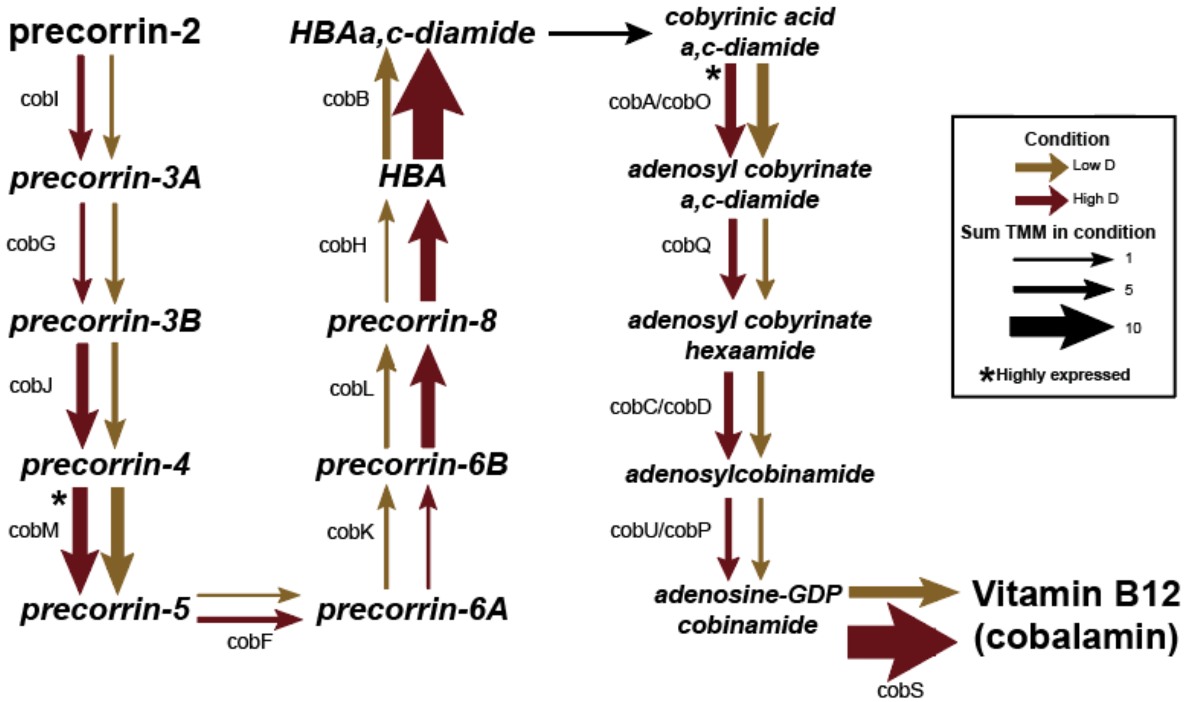

Figure S7 Vitamin B12 synthesis pathway expressed in the burned deep soil. The aerobic cobalamin (Vitamin B12) biosynthesis pathway (adapted from Doxey et al., 2015 and Lu et al., 2020) with arrows indicating the summed TMM in the colored condition.

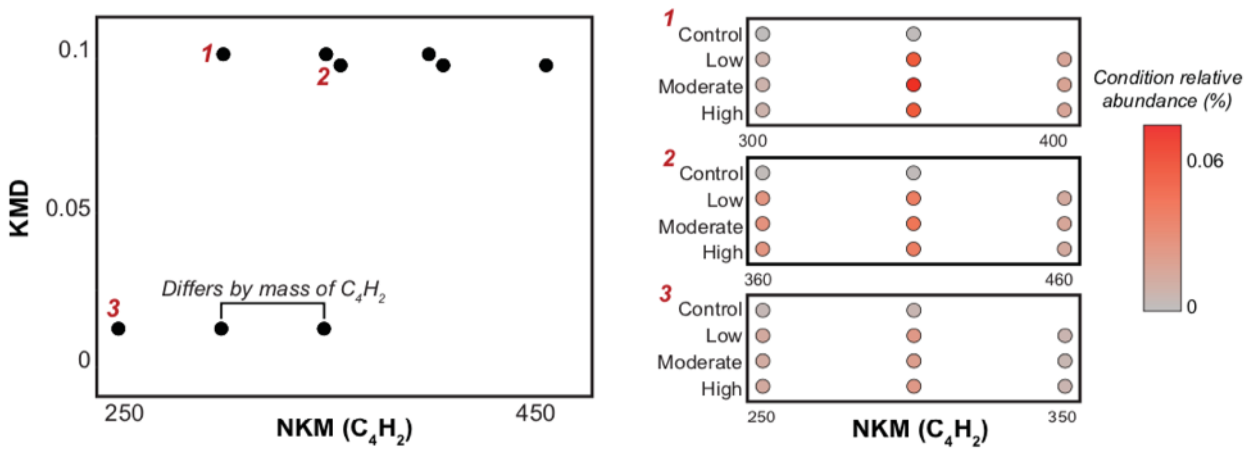

Figure S8 Kendrick mass-defect analyses of FTICR-MS samples. (Left) Example KMD series that differ by the mass of base unit C_4H_2 (Nominal Kendrick Mass; NKM) from samples that span the burn severity gradient. **(Right**) Each of the three series plotted across the burn severity gradient showing how the relative abundance differs and if a new formula is added with increasing severity.

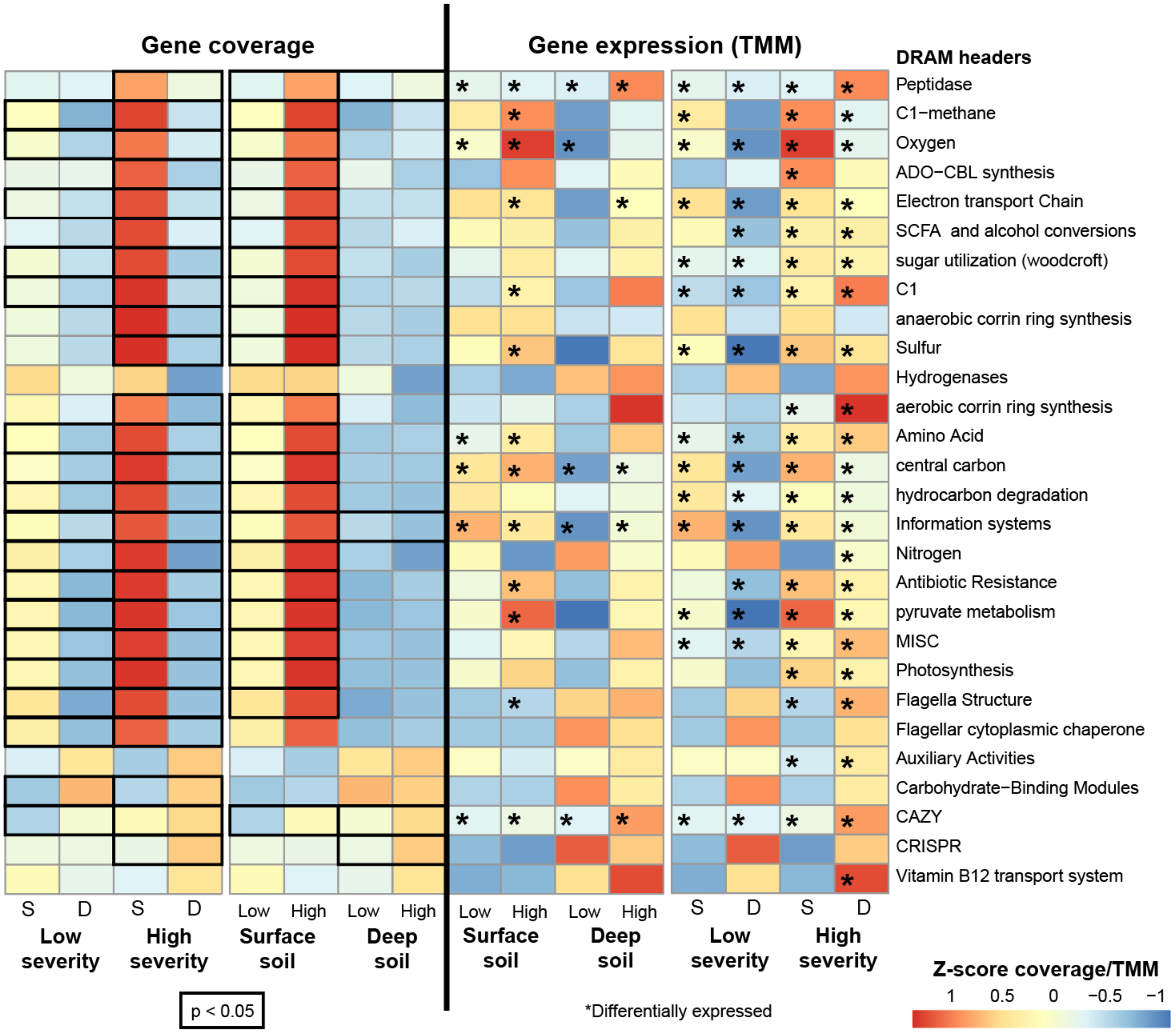

Figure S9 Overall trends between low and high severity surface and deep soils in the gene coverage (left) and gene expression (right) of the different DRAM gene headers. In the gene coverage heat maps, a bolded box indicates that the two conditions are significantly different from one another (pairwise t-test, $p<0.05$). In the gene expression data, stars indicate whether there are genes within that DRAM header that are differentially expressed in that condition relative to the other. Data presented here is included in **Supplementary Data 5**.

Comparison	p-value
Bacterial, surface, control & low	3.7e-5
Bacterial, surface, control & moderate	1.6e-12
Bacterial, surface, control & high	2.6e-13
Bacterial, surface, low & high	1.6e-5
Bacterial, surface, low & moderate	4.9e-5
Fungal, surface, control & low	0.00637
Fungal, surface, control & moderate	2.5e-7
Fungal, surface, control & high	1.5e-8
Fungal, surface, low & high	0.00073
Fungal, surface, low & moderate	0.00637
Bacterial, deep, control & low	8.8e-5
Bacterial, deep, control & moderate	8.3e-5
Bacterial, deep, low & moderate	0.018
Bacterial, deep, low & high	0.018
Fungal, deep, control & moderate	0.0022
Fungal, deep, control & high	0.0173

Table S1. Corresponding p-values for significant differences in bacterial and fungal diversity as shown in **Figure 1e, f**. P-values are from one-sided

Sample	geTMM high expression cutoff
**R85**	1.68
**R86**	3.26
**R89**	1.47
**R90**	3.16
**R93**	1.68
**R94**	3.28
**R109**	1.68
**R110**	2.24
**R113**	1.68
**R114**	2.11
**R117**	1.61
**R118**	1.87

Table S2. The geTMM cutoff values for high expression analysis in each sample. Values correspond to the 20th percentile value TMM. Transcripts were highly expressed if the TMM was above this value for 2/3 samples in any one condition.

Condition	Nodes	Edges	Edge:node	Modules	N samples
**Bacterial communities**					
Control, surface	10426	3662534	351.3	16	16
Low, surface	6234	1194870	191.7	22	24
Moderate, surface	2442	196930	80.6	11	24
High, surface	2193	277073	126.3	11	24
Control, deep	9452	2769191	292.9	16	16
Low, deep	9132	1904471	208.5	21	24
Moderate, deep	5916	1376628	232.7	18	24
High, deep	6415	1151930	179.6	19	24
**Fungal communities**					
Control, surface	2491	271323	108.9	12	16
Low, surface	1548	126698	81.8	11	24
Moderate, surface	238	14366	60.3	2	24
High, surface	260	17026	65.5	2	24
Control, deep	1569	145649	92.8	9	16
Low, deep	681	48389	71.1	5	24
Moderate, deep	242	14558	60.1	3	24
High, deep	374	23626	63.1	3	24

Table S3. Characteristics of WGCNA networks created from 16S rRNA gene sequencing data from each condition.

Guild	# ESVs	% Change with Low	% Change with Mod	% Change with High
Ectomycorrhizal	321	-99.7	-99.9	-99.9
Endophyte	39	-94.2	-95.9	-99.9
Epiphyte	31	-81.1	-99.3	-98.8
Fungal parasite	132	-19.5	-41.1	-65.2
Plant pathogen	528	122.0	24.4	-65.2
Undefined saprotroph	1292	231.0	290.9	298.7
Wood saprotroph	133	-34.2	-99.5	-99.2

Table S4. Percent change in relative abundance from control to low, moderate, and high severity in surface soils of all ASVs assigned to each ecological guild by FUNguild [2]. Data shown here only includes guilds with greater than 30 classified ASVs.

Supplementary Data 1. All sample metadata and associated chemistry data for subsets of samples.

Supplementary Data 2. All metatranscriptomics and metagenomics mapping information including sequencing depth, % reads assembled or binned, and number of MAGs used from each metagenome. This data file also includes all MAG information (completeness, contamination, taxonomy, etc.), their relative abundance across samples, and the selected MAGs of interest for High S and High D.

Supplementary Data 3. Kofamscan HMMs IDs for gene identification used in addition to DRAM and annotation results.

Supplementary Data 4. Metatranscriptomics data including MAG-level geTMM across samples, DRAM-annotated transcript-level geTMM across samples, all differential expression analyses output, and transcript-level geTMM across samples of transcripts from assemblies annotated as inorganic N cycling in DRAM.

Supplementary Data 5. Viral MAG (vMAG) MIUViG information including ID, assembly software, gene count, etc., annotation information of viral AMGs from DRAM, and the output from VirHostMatcher showing each putative MAG-viral linkage with the d2star value.

Supplementary Data 6. Linear discriminant analysis (LDA) output from 16S rRNA gene and ITS amplicon sequencing data, showing fungal and bacterial ASVs that are discriminant for burned or unburned soils.

Supplementary Note 1. Microbiome community compositional shifts with burn

Reflecting observations from prior studies[3–6], bacterial communities in burned soils were characterized by lower diversity and were enriched in Actinobacteria and Bacteroidetes relative to unburned controls (**Extended Data Fig. 2**). The Actinobacteria genera *Arthrobacter, Modestobacter, Blastococcus, and Actinomadura* had the largest increases in relative abundance in burned soils relative to control soils and were discriminant taxa for burned soils (**Supplementary Data 6**). The diversity of fungal communities in surface soils also decreased with fire (**Fig. 1e**) and, similar to previous studies[7–9], shifted from Basidiomycete- to Ascomycete-dominated with the Basidiomycota relative abundance decreasing by ~58% (**Extended Data Fig. 2**). Discriminant fungal taxa included ASVs from the *Sordariomycetes*, *Saccharomycetes*, and *Dothideomycetes*, taxa also found in previous fire studies[6,9] (**Supplementary Data 6**).

The pyrophilous *Ascomycetes* increased in relative abundance by 118% between unburned and burned surface soils (~34% to 75%; **Extended Data Fig. 2**); dominant genera included *Calyptrozyma* (~30% relative abundance in burned surface soil), *Tricharina* (~13%), and *Geopyxis* (~7%). *Geopyxis* has been previously documented as a pyrophilous taxa and is identified as an endophyte, which would aid in their persistence through the fire event and proliferation thereafter[10,11]. The two most abundant *Ascomycete* orders in burned surface soils were *Helotiales* (~31% relative abundance), which have been found in other post-fire soils [12,13] and lab pyrocosm experiments[14] and *Pezizales* (~28%), which is a common post-fire soil

taxa[7,8,15] known for producing resistant structures like spores[16]. The second most dominant *Ascomycetes* family *Pyronemataceae* (~21% relative abundance in burned surface soil) has been found to grow in soils heated in the laboratory to 70°C, to increase in forest soil samples post-fire, and to readily degrade aromatic compounds in the presence of pyrolyzed OM[8,17]. The *Ascomycotes* displayed a higher fire tolerance than the *Basidiomycota*, which had a decrease in relative abundance by ~58% between unburned and burned surface soils, dropping from 50% to 21% relative abundance (**Extended Data Fig. 2**).

Supplementary Note 2. Deeper burned soils are dominated by distinct microbial membership

Only 13 MAGs had an average relative abundance > 0.5% and a standard deviation less than the average relative abundance in High D soils, as compared to the 40 MAGs in High S. This is likely due to the heterogeneity of wildfire impact on deep soils as compared to the homogenizing influence wildfire has on surface soils. These 13 MAGs were affiliated with Actinobacteria, Eremiobacterota, Acidobacteriota, and Proteobacteria (**Supplementary Data 2**). Like the surface soil, the Actinobacteria dominated, with 10/13 of the MAGs being Actinobacteria, representing the genera *Streptomyces*, *SCTD01*, and *Palsa-744*. These 13 MAGs accounted for ~18% of the High D community composition, and 14.3% of total MAG gene expression. The 13 MAGs were also active in Low D samples, albeit to a lesser extent (accounting for ~5% of total expression). Of these 13 MAGs, there were two (RYN_267, RYN_347) that were significantly enriched (pairwise t-test; $p < 0.05$) in High D relative to Low D conditions, representing the Actinobacteria and Eremiobacterota.

Genes associated with thermal resistance were again common in the dominant High D MAGs. Four of these MAGs (R110.16, R86.130, R86.18, R94.45) had metabolic potential to synthesize the environmental stress protectant mycothiol[18]. All MAGs discussed here encoded at least one sporulation gene, and one of the MAGs (RYN_220) was affiliated with the *Streptomyces* which have been shown to form sporogenic structures (aerial hyphae) in response to adverse conditions (i.e., high temperatures, nutrient depletion)[19]. These stress response traits, common to the *Streptomyces*, likely facilitate their dominance on soils one year following high severity wildfire.

Associated with the enrichment (16S data: 155% relative abundance increase from control to High D) and activity of *Streptomyces* in High D samples, we observed high expression of genes encoding the biosynthesis of cobalamin (vitamin B12, *cob* genes), an important coenzyme involved in gene regulation and the synthesis of nucleotides and amino acids. Cobalamin production is conserved within a relatively small group of microorganisms – including *Streptomyces* – and can serve as a keystone function within ecosystems[20]. The entire aerobic cobalamin biosynthesis pathway was expressed in Low D and High D samples (**Supplementary Fig. 7**; pathway adapted[20,21]). Here, a *Streptomyces* MAG (RYN_220) was responsible for 13% of MAG gene expression linked to cobalamin biosynthesis in High D samples. In total, the MAGs mentioned above were responsible for nearly 35% of the total cobalamin biosynthesis MAG gene expression in High D samples. These observations contrast directly with High S samples; the general absence of *Streptomyces* in these samples resulted in limited expression of this pathway. Cobalamin biosynthesis gene expression was ~175% greater in High D samples, and 2 of the aforementioned MAGs (RYN_225, RYN_347) differentially expressed *cobN* and *btuB* (cobalamin transporter gene) in High D samples relative to High S. In the deep soil, the

increased transcription of genes for cobalamin synthesis could be a beneficial consequence of wildfire enriching taxa that encode this trait (i.e., *Streptomyces*). Given the noted importance of this cofactor in mediating a range of critical soil microbiome functions, this process could potentially aid in plant reestablishment[22] and enhance ecosystem function across trophic levels[23].

Supplementary Note 3. Soil chemistry shifts across burn severity and depth

Similar to the microbial data, we found that wildfire had a greater effect on surface soil chemistry (**Supplementary Data 1**). Surface soil pH was 7.8 on average in moderate and high severity plots; both were significantly higher than unburned samples (average pH of 7.2; pairwise t-test, $p<0.05$). Total soil C was significantly lower in the high/moderate (3.3%) than the low (13.3%) and unburned surface soils (15.7%) (pairwise t-test; all burn severity comparisons $p<0.05$). Interestingly, there were no significant changes in DOC, NH_4-N, NO_3-N, or %N, potentially because of small sample sizes. Average DOC was 148 mg/L in control plots, compared to 131, 58 and 43 mg/L in low, moderate, and high severity plots, respectively, though the pattern was not statistically significant. Only NH_4-N had significant changes in deep samples, with significantly higher concentrations in burned (average 0.76 mg/L) vs. unburned (0.19 mg/L) samples (pairwise t-test; $p<0.05$).

Supplementary Note 4. Kendrick mass-defect analyses

Kendrick mass-defect (KMD) analysis with a base unit of C_4H_2 (equivalent to adding benzene to another benzene structure) provides further evidence of linkages between fire severity and increasing polyaromaticity (**Supplementary Fig. 8**). The series reveal the addition of a benzene

ring between unburned and burned soils and an increase in relative abundance of the increasingly polyaromatic formulas (**Supplementary Fig. 8**)

Supplementary Note 5. Networks using Weighted Gene Correlation Network Analysis (WGCNA)

To determine how fire-induced changes in community richness translated into the complexity of potential interactions within the soil microbiome, we performed Weighted Gene Correlation Network Analysis[24] (WGCNA) on the 16S rRNA and ITS gene sequencing data. Across all surface soil samples, we measured strongly decreasing numbers of nodes with increasing burn severity, and associated decreases in the edge-to-node ratio. Similar trends were apparent in the deep soils, albeit with smaller changes between conditions (**Supplementary Table 3**).

Supplementary Note 6. Genetic potential for processing polyaromatics

We additionally looked for genes responsible for degrading naphthalene or removing a ring from a polycyclic aromatic ring (KEGG RM014; **Supplementary Data 3**) and found that none of the MAGs encoded a naphthalene 1,2-dioxygenase (nahA), but 33 MAGs encoded 2-hydroxychromene-2-carboxylate isomerase (EC:5.99.1.4, nahD) and 10 encoded trans-o-hydroxybenzylidenepyruvate hydratase-aldolase (EC:4.1.2.45, nahE). These two genes were also expressed in the dataset, but only by two MAGs in the *Proteobacteria*.

Supplementary Note 7. Broad functional shifts with burn severity

To analyze broad shifts in functional potential and gene expression between Low and high severity conditions within both soil depths, we categorized genes using DRAM gene headers.

There were 885,498 genes annotated with DRAM in the metagenomic dataset and 146,894 transcripts annotated with DRAM that had counts of at least 10 across all 12 samples. A DESeq2 analysis of the DRAM-annotated transcripts revealed 352 differentially expressed genes between Low S and High S samples and 26 between Low D and High D samples (**Supplementary Data 4**). Most of the differential expression in High S and High D was attributed to the Actinobacteria phyla (97.9% and 50% of differentially expressed genes, respectively). There were genera and family-level differences between the activity in both soil depths, with *Actinobacteriota* genera *Blastococcus* (69.1%), *SCTD01* (10.4%) *Arthrobacter* (4.8%) and *Modestobacter* (2.7%) as the large players in High S and the family *Streptosporangiaceae* (35%) being the biggest player in High D. Outside of the *Actinobacteriota* phylum, the *Proteobacteria* genus *Palsa-1478* was responsible for 45% of differentially expressed genes in High D samples. Thus, the metatranscriptomics data additionally reflects the increased susceptibility of surface soil to fire relative to deep soils.

There was a large change in broad functional potential between Low S and High S samples; of the 28 DRAM gene headers analyzed, 22 were significantly different in coverage between the severity conditions (**Supplementary Fig. 9**). Though there is a large shift in microbiome functional potential, the metatranscriptomics data reveals that there is less of a change in gene expression between Low S and High S conditions, exhibiting potential functional redundancy. Thirteen DRAM gene headers have differentially expressed genes, and 7 of these were genes only differentially expressed in high severity conditions (**Supplementary Fig. 9**). In total, there were 288 genes differentially expressed in High S and 64 genes differentially expressed in Low S. Reflecting the large role of *Actinobacteriota* in high severity-impacted soils mentioned above,

the majority of differentially expressed genes in High S were from the *Actinobacteriota* phyla (97.9% of differentially expressed genes). Specifically, the *Actinobacteriota* genera *Blastococcus* (69.1%), *SCTD01* (10.4%) *Arthrobacter* (4.8%) and *Modestobacter* (2.7%)

Contrastingly, the microbiome of deep soils exhibited a much smaller shift in functional potential with only 4/28 DRAM gene headers having a significant change in coverage between Low D and High D (peptidase, information systems, CAZY, and CRISPR; **Supplementary Fig. 9**). There was also little change in gene expression; there were 6 DRAM headers (26 total genes) with differentially expressed genes, 20 that were differentially expressed in High D and 6 in Low D (**Supplementary Fig. 9**). The *Actinobacteriota* phyla was responsible for the largest proportion of differentially expressed genes following high-severity fire (50%) and the family *Streptosporangiaceae* and was responsible for 35% of the differentially expressed genes. The *Proteobacteria* genera *Palsa-1478* was also responsible for a large amount of differentially expressed genes (45%).

Supplementary Note 8. Fungal MAGs encode secondary metabolite clusters

The *Coniochaeta* MAG (R110-5) encoded 26 secondary metabolite clusters, including 12 PolyKetide Synthase (PKS) clusters and 4 Non-Ribosomal Peptide Synthetase (NRPS) clusters. This trait appears to be conserved across the *Coniochaeta* genera, as the other four *Coniochaeta* genomes deposited on JGI MycoCosm encode an average of ~36, ranging from 25-66. Both polyketide and non-ribosomal peptide secondary metabolites display a diverse array of biological uses and properties including antibiotic synthesis[25], siderophore production[26], and fungal developmen[27] (e.g., sporulation factors), and may give organisms a competitive edge, especially

under stressful conditions. The other fungal MAG (R113-184; *Leotiomycetes*) may persist following wildfire due to an endophytic lifestyle; 36 *Leotiomycetes* ESV's were classified as endophytes by FUNGuild. Pyrophilous endophytic fungi have been found to occur survive fire events in small-scale refugia then, triggered by the fire, produce reproductive sporocarps [10].

References

1. Parson, A., Robichaud, P. R., Lewis, S. A., Napper, C. & Clark, J. T. *Field guide for mapping post-fire soil burn severity*. *USDA Forest Service - General Technical Report RMRS-GTR* (2010). doi:10.2737/RMRS-GTR-243
2. Nguyen, N. H. *et al.* FUNGuild: An open annotation tool for parsing fungal community datasets by ecological guild. *Fungal Ecol.* **20**, 241–248 (2016).
3. Fernández-González, A. J. *et al.* The rhizosphere microbiome of burned holm-oak: potential role of the genus Arthrobacter in the recovery of burned soils. *Sci. Rep.* **7**, 6008 (2017).
4. Villadas, P. J. *et al.* The Soil Microbiome of the Laurel Forest in Garajonay National Park (La Gomera, Canary Islands): Comparing Unburned and Burned Habitats after a Wildfire. *Forests* **10**, 1051 (2019).
5. Weber, C. F., Lockhart, J. S., Charaska, E., Aho, K. & Lohse, K. A. Bacterial composition of soils in ponderosa pine and mixed conifer forests exposed to different wildfire burn severity. *Soil Biol. Biochem.* **69**, 242–250 (2014).
6. Whitman, T. *et al.* Soil bacterial and fungal response to wildfires in the Canadian boreal forest across a burn severity gradient. *Soil Biol. Biochem.* **138**, 107571 (2019).
7. Pulido-Chavez, M. F., Alvarado, E. C., DeLuca, T. H., Edmonds, R. L. & Glassman, S. I.

High-severity wildfire reduces richness and alters composition of ectomycorrhizal fungi in low-severity adapted ponderosa pine forests. *For. Ecol. Manage.* **485**, 118923 (2021).

8. Reazin, C., Morris, S., Smith, J. E., Cowan, A. D. & Jumpponen, A. Fires of differing intensities rapidly select distinct soil fungal communities in a Northwest US ponderosa pine forest ecosystem. *For. Ecol. Manage.* **377**, 118–127 (2016).
9. Yang, T. *et al.* Distinct fungal successional trajectories following wildfire between soil horizons in a cold-temperate forest. *New Phytol.* **227**, 572–587 (2020).
10. Raudabaugh, D. B. *et al.* Where are they hiding? Testing the body snatchers hypothesis in pyrophilous fungi. *Fungal Ecol.* **43**, 100870 (2020).
11. Vrålstad, T., Holst-Jensen, A. & Schumacher, T. The postfire discomycete Geopyxis carbonaria (Ascomycota) is a biotrophic root associate with Norway spruce (Picea abies) in nature. *Mol. Ecol.* **7**, 609–616 (1998).
12. Day, N. J. *et al.* Wildfire severity reduces richness and alters composition of soil fungal communities in boreal forests of western Canada. *Glob. Chang. Biol.* **25**, 2310–2324 (2019).
13. Meng, M., Wang, B., Zhang, Q. & Tian, Y. Driving force of soil microbial community structure in a burned area of Daxing'anling, China. *J. For. Res.* (2020). doi:10.1007/s11676-020-01229-0
14. Bruns, T. D., Chung, J. A., Carver, A. A. & Glassman, S. I. A simple pyrocosm for studying soil microbial response to fire reveals a rapid, massive response by Pyronema species. *PLoS One* **15**, e0222691 (2020).
15. Dove, N. C. & Hart, S. C. Fire reduces fungal species richness and in situ mycorrhizal colonization: A meta-analysis. *Fire Ecol.* **13**, 37–65 (2017).

16. Baar, J., Horton, T. R., Kretzer, A. M. & Bruns, T. D. Mycorrhizal colonization of Pinus muricata from resistant propagules after a stand-replacing wildfire. *New Phytol.* **143**, 409–418 (1999).

17. Kipfer, T., Egli, S., Ghazoul, J., Moser, B. & Wohlgemuth, T. Susceptibility of ectomycorrhizal fungi to soil heating. *Fungal Biol.* **114**, 467–472 (2010).

18. Newton, G. L., Buchmeier, N. & Fahey, R. C. Biosynthesis and Functions of Mycothiol, the Unique Protective Thiol of Actinobacteria. *Microbiol. Mol. Biol. Rev.* **72**, 471–494 (2008).

19. McCormick, J. R. & Flärdh, K. Signals and regulators that govern Streptomyces development. *FEMS Microbiol. Rev.* **36**, 206–231 (2012).

20. Lu, X., Heal, K. R., Ingalls, A. E., Doxey, A. C. & Neufeld, J. D. Metagenomic and chemical characterization of soil cobalamin production. *ISME J.* **14**, 53–66 (2020).

21. Doxey, A. C., Kurtz, D. A., Lynch, M. D. J., Sauder, L. A. & Neufeld, J. D. Aquatic metagenomes implicate Thaumarchaeota in global cobalamin production. *ISME J.* **9**, 461–471 (2015).

22. Palacios, O. A., Bashan, Y. & De-Bashan, L. E. Proven and potential involvement of vitamins in interactions of plants with plant growth-promoting bacteria—an overview. *Biol. Fertil. Soils* **50**, 415–432 (2014).

23. Akduman, N. *et al.* Bacterial vitamin B12 production enhances nematode predatory behavior. *ISME J.* **14**, 1494–1507 (2020).

24. Langfelder, P. & Horvath, S. WGCNA: an R package for weighted correlation network analysis. *BMC Bioinformatics* **9**, 559 (2008).

25. Vining, L. C. Functions of Secondary Metabolites. *Annu. Rev. Microbiol.* **44**, 395–427

(1990).

26. Kramer, J., Özkaya, Ö. & Kümmerli, R. Bacterial siderophores in community and host interactions. *Nat. Rev. Microbiol.* **18**, 152–163 (2020).

27. Calvo, A. M., Wilson, R. A., Bok, J. W. & Keller, N. P. Relationship between Secondary Metabolism and Fungal Development. *Microbiol. Mol. Biol. Rev.* **66**, 447–459 (2002).
