# Supplementary material for: Wildfire-dependent changes in soil microbiome diversity and function
Source: Nat Microbiol. 2022 Aug 25;7(9):1419–30. doi: 10.1038/s41564-022-01203-y (PMC9418001; doi:10.1038/s41564-022-01203-y)
Supplement: Supplementary file 2 — Reporting Summary [file 41564_2022_1203_MOESM2_ESM.pdf]

nature portfolio

Corresponding author(s): Michael J. Wilkins

Last updated by author(s): Jul 13, 2022

Reporting Summary

Nature Portfolio wishes to improve the reproducibility of the work that we publish. This form provides structure for consistency and transparency in reporting. For further information on Nature Portfolio policies, see our Editorial Policies and the Editorial Policy Checklist.

Statistics

For all statistical analyses, confirm that the following items are present in the figure legend, table legend, main text, or Methods section.

n/a	Confirmed	
☐	☒	The exact sample size (*n*) for each experimental group/condition, given as a discrete number and unit of measurement
☐	☒	A statement on whether measurements were taken from distinct samples or whether the same sample was measured repeatedly
☐	☒	The statistical test(s) used AND whether they are one- or two-sided<br>*Only common tests should be described solely by name; describe more complex techniques in the Methods section.*
☒	☐	A description of all covariates tested
☐	☒	A description of any assumptions or corrections, such as tests of normality and adjustment for multiple comparisons
☐	☒	A full description of the statistical parameters including central tendency (e.g. means) or other basic estimates (e.g. regression coefficient) AND variation (e.g. standard deviation) or associated estimates of uncertainty (e.g. confidence intervals)
☐	☒	For null hypothesis testing, the test statistic (e.g. *F*, *t*, *r*) with confidence intervals, effect sizes, degrees of freedom and *P* value noted<br>*Give P values as exact values whenever suitable.*
☒	☐	For Bayesian analysis, information on the choice of priors and Markov chain Monte Carlo settings
☒	☐	For hierarchical and complex designs, identification of the appropriate level for tests and full reporting of outcomes
☐	☒	Estimates of effect sizes (e.g. Cohen's *d*, Pearson's *r*), indicating how they were calculated

Our web collection on statistics for biologists contains articles on many of the points above.

Software and code

Policy information about availability of computer code

Data collection	No software was used for the collection of data (e.g., chemistry, raw sequencing reads).
Data analysis	Bioinformatic tools used for sequencing data processing and analysis: QIIME2 (2018.11), DADA2, FUNGuild (v1.2), BBduk, Sickle (v1.33), MEGAHIT (v1.2.9), MetaBAT2 (v2.12), checkM (v1.1.2), GTDB-Tk (V1.3.0), dRep (v2.2.3), CoverM (v0.6.0), gRodon, OrthoFinder (v2.5.4), MAFFT, TrimA1 (v1.4.rev22), iqtree (v1.6.9), FigTree (1.4.4), BUSCO (v4.0.6), CEGMA, RnaSPAdes (v3.13.0), DRAM (v1.0), HMMER, BBMap (v38.70), HTSeq, DESeq2, edgeR, VIRSorter2 (v2.2.2), CyVerse ClusterGenomes (v1.1.3), vConTACT2 (v0.9.8), Dram-v (v1.2.0), Geneious (v2020.0.3), CRisprASSembler (v1.0.1), VirHostMatcher (v1.0.0). The following tools were used for data processing and visualization: RStudio (v3.6.1), vegan (v2.5-7), phyloseq (v.1.28.0), Adobe Illustrator 2020 (v25.2). The following tools were used to process FTICR-MS data: ftmsRanalysis, Formularity, Bruker Daltonics DataAnalysis (version 4.2).

For manuscripts utilizing custom algorithms or software that are central to the research but not yet described in published literature, software must be made available to editors and reviewers. We strongly encourage code deposition in a community repository (e.g. GitHub). See the Nature Portfolio guidelines for submitting code & software for further information.

Data

Policy information about availability of data

All manuscripts must include a data availability statement. This statement should provide the following information, where applicable:

- Accession codes, unique identifiers, or web links for publicly available datasets
- A description of any restrictions on data availability
- For clinical datasets or third party data, please ensure that the statement adheres to our policy

The data availability statement includes the NCBI Bioproject to assess the metagenomic and metatranscriptomic reads, bacterial and viral MAGs, 16S rRNA gene sequencing reads, and ITS amplicon reads (PRJNA682830). The two fungal MAGs are deposited in the JGI MycoCosm portal and can be assessed at https://mycocosm.jgi.doe.gov/ColoR113_1 and https://mycocosm.jgi.doe.gov/ColoR110_1. The raw FTICR-MS data is deposited on Zenodo with identifier doi:10.5281/zenodo.5182305. All data is publicly accessible at time of initial submission. The following databases were also used: Silva (release 132), UNITE (v8.3), GTDB-Tk (v1.3.0).

Human research participants

Policy information about studies involving human research participants and Sex and Gender in Research.

Reporting on sex and gender	N/A to this study.
Population characteristics	N/A to this study.
Recruitment	N/A to this study.
Ethics oversight	N/A to this study.

Note that full information on the approval of the study protocol must also be provided in the manuscript.

Field-specific reporting

Please select the one below that is the best fit for your research. If you are not sure, read the appropriate sections before making your selection.

☐ Life sciences ☐ Behavioural & social sciences ☒ Ecological, evolutionary & environmental sciences

For a reference copy of the document with all sections, see nature.com/documents/nr-reporting-summary-flat.pdf

Ecological, evolutionary & environmental sciences study design

All studies must disclose on these points even when the disclosure is negative.

Study description	To assess the influence of wildfire burn severity on the soil microbiome, we used coupled microbial approaches to comprehensively characterize the soil microbiome across a burn severity gradient (unburned control, low, moderate, and high severity). With marker gene approaches (16S rRNA gene sequencing, ITS amplicon sequencing), we showed that deeper mineral horizon soils were more insulated from the impacts of wildfire and that wildfire exerted a homogenizing influence on the organic horizon microbiome, with Actinobacteria dominating the post-fire soils. Metagenomic and metatranscriptomic sequencing coupled with FTICR-MS analyses within low and high severity-impacted soils showed the active degradation of fire-derived aromatic soil organic matter compounds in burned soils. This approach also allowed us to identify DNA and RNA viral genomes (vMAGs) within the post-fire system, with evidence of active viral predation of dominant and active MAGs. This publicly-available dataset offers a myriad of opportunities for future research into the impact of wildfire on the soil microbiome, which is becoming increasingly important to understand as wildfires increase in frequency, severity, and duration across the globe.
Research sample	Depth-resolved soil samples (organic and mineral horizon) from unburned, low, moderate, and high severity-impacted conditions were used for all data collection. A total of 176 samples were collected with a large amount of replication to fully capture the heterogeneity inherent in samples. For metagenomics and metatranscriptomics analyses, we used triplicate of each condition.
Sampling strategy	Four candidate burn severity gradients were selected based on US Forest Service, Burned Area Emergency Response program (BAER) remotely sensed imagery and maps, and subsequently field validated. Aspect, slope, and elevation were recorded at each sampling plot and was kept generally consistent across all plots. Each gradient comprised low, moderate, and high severity sites and an unburned control. Low, moderate, and high severity sites had >85%, 20-85%, and <20% surficial organic matter cover, respectively. Samples were collected on August 16 and 19 of 2019, approximately one year following containment of both fires. At each sampling site, a 3 m x 5 m sampling grid with six m2 subplots was laid out perpendicular to the dominant slope (Figure S1). Subsamples of the organic soil horizon (i.e., litter and duff; O-horizon) and upper mineral soil horizon (0-5 cm; A-horizon) were collected with a sterilized trowel in each subplot for DNA and RNA extractions and subsequent microbial analyses. In three subplots, additional material was collected for chemical analyses. Samples for RNA analyses were immediately flash-frozen using an ethanol-dry ice bath and subsequently placed on ice to remain frozen in the field. Samples for DNA extractions and chemical analyses were immediately

	placed on ice and all samples were transported to the laboratory at Colorado State University (CSU). Soils for DNA and RNA extractions were stored at -80°C in the laboratory until processing.
Data collection	ARN led the majority of data collection and completed all DNA and RNA extractions, and water extractions and SPE for FTICR-MS. 16S rRNA genes in extracted DNA were amplified and sequenced at Argonne National Laboratory on the Illumina MiSeq. ITS amplicon amplification and sequencing was completed at the University of Colorado BioFrontiers Institute Next-Gen Sequencing Core Facility on the Illumina MiSeq platform. Data was processed by ARN using QIIME and the Silva and Unite databases.<br>A FTICR-MS located at the Environmental Molecular Sciences Laboratory in Richland, WA, was used to collect DOM high-resolution mass spectra of all DOM extracts and the Formularity software was used to assign formulas to peaks. The FTICR-MS data collection was completed by RKC.<br>Metagenomics and metatranscriptomics sequencing on the 12 subsetted samples was completed at Genomics Shared Research at the Colorado Cancer Center. See methods for sequencing details. Bioinformatics tools were utilized by ARN to process the raw reads. Fungal MAGs were annotated by ASS, SJM, IVG, and AS.
Timing and spatial scale	Soils were collected at one time point one year following the containment of the Ryan and Badger Creek Wildfires. To characterize spatial heterogeneity, we collected samples across two ~200 m burn severity gradient transects within each fire.
Data exclusions	None of the aforementioned collected data was excluded from this study.
Reproducibility	Analyses were performed across biological replicate samples. Furthermore, biological trends (i.e., shifts in community composition) are inferred from several different analytical methods
Randomization	Sample allocation was not random and all samples were treated as equal as possible. Metagenome library construction was randomized, although samples were not blinded. FTICR MS analyses were randomized at EMSL
Blinding	DOM analyses via FTICR MS were performed using blinding.

Did the study involve field work? ☐ Yes ☐ No

Field work, collection and transport

Field conditions	Sampling was conducted in lodgepole pine (Pinus contorta) forests burned by Badger Creek (8215 ha) and Ryan (11567 ha) fires during 2018 in the Medicine Bow National Forest. There were no precipitation events during the time of sampling.
Location	Coordinates for all sampling plots can be found in Supplementary Data 1.
Access & import/export	We did not have to obtain any permits or import/export any sample material.
Disturbance	Small volumes of soil were collected within burn scars, and as such any disturbances were extremely minimal.

Reporting for specific materials, systems and methods

We require information from authors about some types of materials, experimental systems and methods used in many studies. Here, indicate whether each material, system or method listed is relevant to your study. If you are not sure if a list item applies to your research, read the appropriate section before selecting a response.

Materials & experimental systems

n/a	Involved in the study
☒	☐ Antibodies
☒	☐ Eukaryotic cell lines
☒	☐ Palaeontology and archaeology
☒	☐ Animals and other organisms
☒	☐ Clinical data
☒	☐ Dual use research of concern

Methods

n/a	Involved in the study
☒	☐ ChIP-seq
☒	☐ Flow cytometry
☒	☐ MRI-based neuroimaging
